# An Estimate of the Effects from Precision Livestock Farming on a Productivity Index at Farm Level. Some Evidences from a Dairy Farms’ Sample of Lombardy

**DOI:** 10.3390/ani10101781

**Published:** 2020-10-01

**Authors:** Felicetta Carillo, Fabio Abeni

**Affiliations:** 1 CREA—Research Centre for Agricultural Policies and Bioeconomy, 00198 Rome, Italy; felicetta.carillo@crea.gov.it; 2 CREA—Research Center for Animal Production and Aquaculture, 26100 Lodi, Italy

**Keywords:** precision livestock farming, livestock productivity, treatment effect model

## Abstract

**Simple Summary:**

The adoption of digital technologies and sensors at farm level can give several benefits in terms of animal welfare, environmental sustainability, and farmer’s quality of life; but the evaluation of possible productive returns is still the most critical aspect. At the same time, it is very difficult to plan a controlled contemporary study with a dairy farm that involves adopting or not adopting the different technologies today available, and measure without any bias their productive performance at herd level. This paper used the results of a planned survey in dairy farms in Lombardy (Italy) and tries to quantify if there is a gap in productivity between farms with different adoption degree of digital technologies in herd management. Through a statistical two-stage treatment regression technique, the study was able to find a positive relationship between technology adoption and herd productivity.

**Abstract:**

This paper aimed at verifying if and to what extent the use of information technologies for dairy farming positively affects productivity of farmed herd. To do this we estimated the effects of precision farming on a productivity index at herd level, utilizing individual farms data of about 500 livestock farms. Farms are specialized in bovine milk production and are localized in Lombardy, that is one of the most important areas of Italian dairy farming. Using a two-stage treatment regression model, to solve the selection bias due to both observed and un-observed individual heterogeneity in the technology adoption, the study found a positive relationship between adopter status and the proxy of herd productivity.

## 1. Introduction

The main future challenge faced by the agriculture industry is how to increase production in order to respond to the growth in food demand, while preserving the natural resources and environment. Particularly, for protein products of animal origin, it will be increasingly important to introduce innovations that contribute to the improvement of productivity of such productive systems in a sustainable manner. The productivity of dairy livestock has grown a lot in recent years at the European level. The average yield in the past was mainly driven by improvements in the individual animal (genetics) and production process (introduction of innovations on animal nutrition, etc.) and reflected significant changes in the structure of the sector (farms that remain on the market are those who are the biggest in size terms and the most efficient). Currently, improving farm-level efficiency appears to be the main way to further increase the sector productivity. Generally, digital and geospatial technologies to monitor, assess, and manage soil, climatic and genetic resources, allow to balance the economic, environmental, and social dimensions of sustainable food production [1]. Also, the precision livestock farming (PLF) tools are widely considered as technologies that potentially could determine efficiency improvements of individual farms, contextually having positive effects on animal health and welfare.

In general, precision farming tools help farmers to improve input allocation decisions, by which lower production costs and/or increase outputs [2]. Specifically, PLF is a managing strategy, using information and communication technology (ICT) to monitor and inform farmer about physiological or behavioral parameters on individual cows or groups of cows, that are related to some events (health, estrous, rumination, production levels and quality of milk, etc.) which require action by the farmer, allowing a more efficient management of herds. Several automatic detection devices are used in practice to improve cows’ management. For example, sensor systems are used to more precisely formulate the diet and food ration for specific animals or groups of them [3], while sensor and alarm systems are adopted to identify cows’ estrous and consequently the more appropriate time for insemination [4].

The majority of specific literature showed that the information gathered in this way is quite useful in assisting farmers in making input allocation and herd management decisions, that are better than it would be with conventional management practices [5]. However, little information currently exists about the relative magnitude of the economic and productivity consequences of the adoption of digital detection systems for farming. Moreover, the empirical studies often showed conflicting results suggesting that there is uncertainty in returns to investment and a lack of demonstrated effects of these technologies on yields, input-use and economic performance [6,7]. To our knowledge, there are no specific studies on the effects of early experience with a specific technology and the long-time farmer attitude for its use. However, as remembered by Stone (2020) [8], trialability and observability are two characteristics required by the farmer before considering a possible investment in innovations. These differences in signals about the convenience for the farmers to adopt such technologies are shown, as potential benefits of such technologies widely depend on farms and farmer characteristics [2,6]. For example, they strongly depend on the dimension of herd (productive scale), but also from the ability of the farmer to effectively realize these potential advantages through an appropriate use of the tools. This may be attained in time by the acquisition of specific skills by the farmer and his personnel. Particularly, as these technologies are constantly evolving, and they require the intervention of the farmer, training is an important issue to effectively use the information provided by the herd management system more efficiently. Then, it is not a trivial result to verify if and to what extent farms actually gain the potential benefits from using these systems. Moreover, to the best of our knowledge, no empirical research was conducted on the Italian livestock system.

Prevention of clinical disease due to a better peripartum management of the cow may prevent most of the clinical diseases in the first 21 d of lactation, which may cause milk, fat, and protein losses [9] that have an important economic impact in a systems, like that in Po Valley, where milk payment is based on its quality. A good example about the relationship between animals’ welfare monitoring and the productive and economic impact of PLF can be represented by the prevention of metabolic disorders like ketosis and its subclinical form. Actually, the possible in-line milk testing for ketone bodies allows to quickly detect subclinical signs of ketosis that, according to Steeneveld et al. (2020) [10], causes significant milk yield losses which represent the highest contribution to the costs of a single case, whereas a clinical ketosis implies also a veterinary treatment cost that may lead to a global cost per case of on average € 709.

Whether PLF technologies can improve or not the productive level of dairy farms, after their introduction, is not a consolidated result because this could be affected by other several changes related, for example, to the training of farm personnel as well as to other management issues typical of a transition period. This might affect a simple evaluation carried comparing the same farms just before and just after the technology adoption. The aim of this paper is to introduce an alternative approach with a two stages regression as a reasonable way to by-pass the problem from a time series evaluation of the changes from PLF adoption.

## 2. Materials and Methods 

As we said before, the study wanted to estimate the differentials of productivity between those farms adopting of PLF technology and those who are non-adopters. To do this, we predicted an average productivity index at the herd level, with a dummy variable representing PLF adoption, trying to keep fixed the other factors that could influence the productivity measure used. The herd productivity index was the 305-d average milk yield per cow performance for each herd.

Overall, PLF adoption helps to better manage the herd, and, more specifically, the several types of sensors used by sample farms could be grouped in: (a) Sensors to detect the estrum for an efficacy reproduction; (b) sensors for milk production and quality controls; and (c) sensors for timely and effective livestock disease surveillance. These different management factors, as proved in the specific literature, have a direct effect on the chosen yield productivity measure. For example, health problems affect milk production in various ways for each kind of disease [9], even at the subclinical level, as was demonstrated for mastitis [11], ketosis [12], and lameness [13]. Moreover, milking monitoring allows to rapidly adequate the diet to the productive ability of each cow or group of them. Lastly, reproductive management is the basis to attain milk production as well as a replacement herd to gain the genetic value of lactating herd.

On the other hand, herd productivity could depend on many other factors, among which three main categories can be distinguished: Natural resource endowments (such as races and genetics, soil fertility and climate), technologies (machineries, stables equipment, etc.), techniques of herd management (the housing conditions, daily times of cows milked, etc.). 

In order to isolate these effects on our productivity variable and to measure the differences due to the management efficiency of herd, net of other influencing components, we tried to keep the other non-observed factors fixed. In other words, we tried to restrict the analysis to a “no-interference” setting. To do this we adopted different empirical strategies.

First of all, we carried out interviews in an area that is highly homogeneous from a production point of view, specifically as regards milking technologies, the main techniques used to manage the herd (housing conditions, types and composition of feeds, daily times of cows milked, etc.) and the natural capital (type of races, genetic improvement level) used by livestock farms [14].

Secondly, to reduce estimations bias due to the self-selection problem, we used a linear regression with endogenous treatment effects and other covariates to control for farm productive scale. 

**Figure 1:**
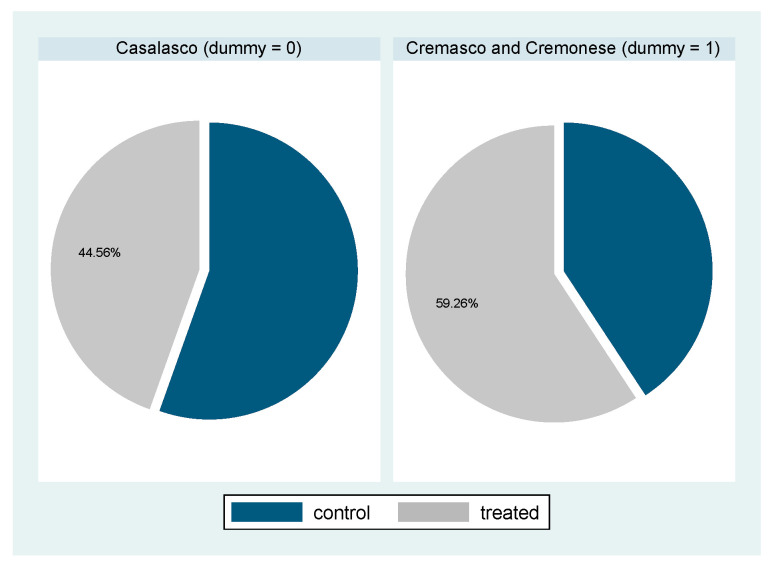
Percentage of localization areas adoption. Source: Our elaborations on survey.

Data were collected through a survey conducted during the year 2016 submitted to a sample of 490 dairy livestock farms located in the Cremona administrative province. In this area, the milk livestock sector is of economic importance and a massive presence of large farms typify the productive structure of sector, which averagely have about 200 cattle farmed. Specifically, the interviewed farms are located in three homogeneous areas from a production point of view: The Cremasco area, the Casalasco area, and the Cremonese area. The sample represented the 82% of the dairy farms in the province of Cremona [15] at the time of the survey being carried out. The farm enrolment was randomized within each geographical area, with the only constraint being to cover all the municipality within the area with at least one farm, following the usual monthly intervention schedule of each technician for the APA routine work for the official production test of each herd. The final sample was composed of 234 farms using at least one sensor system, the adopters’ group, and 256 farms which did not adopt such technology, the control group. Specifically, as shown in Figure 1, the Cremasco and Cremonese areas have an adoption rate of about 60%, while in the Casalasco area the adopters represent less than 45% of sample.

The empirical approach used was the estimation of an “adoption gain”, that could derive from the use of several sensor systems to support herd management, calculated as differences in the herd productivity index. Methodologically, using a two-stage treatment regression model, we estimated the average treatment effect on treated (ATET), that measures the difference in mean outcomes between units (farms) assigned to the treatment (technology adopters, in our case) and units (farms) assigned to the control (non-adopters).

A two-stage regression technique allowed us to estimate an average treatment effect and other parameters of a linear regression model augmented with endogenous binary-treatment variable. This technique reduces the probable bias of estimations due to the self-selection problem. The bias could derive from the presence of some farms’ characteristics that induce farmers self-selection into the choice of technology adoption and are also correlated with outcomes (Y). In general, in a non-randomized setting it is essential to identify the treatment effect, considering the different pre-treatment conditions between treated and untreated [16]. Such specific characteristics of farmers may or not may be observed and both confounds the non-experimental setup. In our case, for example, as younger farmer tends to choose to adopt new technologies, the decision could be endogenous and, then, it must be explicitly modelled by a set of observable characteristics. 

More formally, the endogenous treatment-regression model is composed of an equation for the outcome *y_j_* and an equation for the endogenous treatment *t_j_*,

(1) $$y_{i\;}=\;x_{j}\beta +\;\delta t_{j}+\epsilon_{j}$$

(2) $$t_{j}=\;\{\begin{array}{c} 1,\;\mathrm{if}\;w_{j}\gamma +u_{j}>0\\ 0,\;\mathrm{otherwise}\; \end{array}$$

where *x_j_* are the covariates used to model the outcome, *w_j_* are the covariates used to model treatment assignment, and the error terms *ε_j_* and *u_j_* are bivariate normal with mean zero and covariance matrix

(3) $$\left[\begin{array}{cc} \sigma^{2} & \rho \sigma \\ \rho \sigma & 1 \end{array}\right].$$

The covariates *x_j_* and *w_j_* are unrelated to the error terms; in other words, they are exogenous. 

Technically, by running the official STATA (version 16.1) command called etregress, with option twosteps, whose estimation is based on the [17] Heckman selection model, we got the following outputs.

The first stage procedure performed a probit model (treatment equation) to estimate the likelihood that farm adopts sensor technologies, using as Y variable a dichotomous taking on value 1 if at least one sensor system is present in the farm and 0 otherwise. For this equation, as independent variables we used the localization of farm, the farm size and the age of farmer, to model the characteristics that influence the choice of adoption. We emphasize that our choice of using these regressors is widely supported by most of literatures, which have explored on the adoption process of such innovations [18]. Particularly some scholars evidenced the young age of farmers as key determinant, showing that the youngest are more sensitive to invest in ICT technologies [6]. Following these evidences, we use a categorical variable that represent group of farmers’ ages as shown in a Table 1. Moreover, as in general the same localization generates imitative behaviors of firms in the adoption of technology [18], we use a variable representing those areas that have a higher adoption rate than average. So, we use a dummy variable, that assume value 1 if farm is localized in the Cremasco or Cremonese areas and 0 otherwise.

Larger farms are also hypothetically more likely to adopt a new technology and specifically farm size was identified as the most important aspect influencing the adoption of precision farming technologies [19]. However, we alternatively used two proxies to represent the farm size, the total hectares of used agricultural area (UAA) and the total unit of workers, but they are not statistically significant. 

In the second stage the STATA command regresses the main equation, evaluating the adoption effect on a proxy of farm’s productivity. As already said, the productivity index (Y) is the tons of milk averagely produced by one cow in a year, taken in their natural logarithm. For this equation our interest variable is the dichotomous one, representing PLF adoption, whose coefficient was estimated by the treatment equation. Moreover, to take also account the scale effect on productivity level, we have included two variables as covariates, the herd size and its square, aiming to capture heterogeneity in the scale effect.

## 3. Results

Table 1 and Table 2 show some descriptive statistics of the variables used in the model. As we can see in the Table 1, the whole sample was composed by farms that were averagely of large size, being equipped with about 96 hectares of UAA, with a little more than 4 work units and with about 189 cows in lactation. However, the adopters’ group was larger than average, having an UAA size of about 122 hectares, a little less than 5 work units and more than 230 cows in the herd, confirming the heterogeneity of characteristics of the comparison groups (Table 1) that we used in the treatment regression to correct the estimates. 

**Figure 2:**
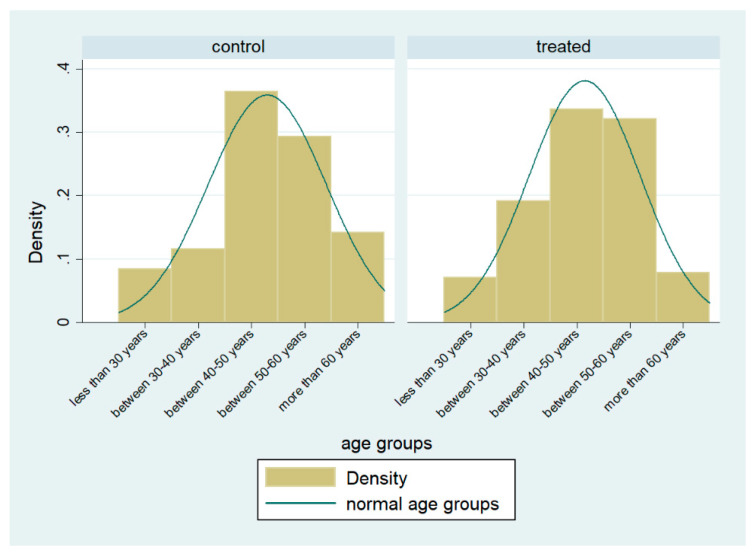
Percentage of age groups for treated and control groups. Source: Our elaborations on survey.

Regarding the age of farmers, as we can better see with the Figure 2, the treated group has a distribution closer to a normal one, with a higher percentage of farmers aged between 30 and 40 and, while a lower percentage of those who are older than 60 years, supporting our choice of using age as variable to predict probability of adoption.

**Figure 3:**
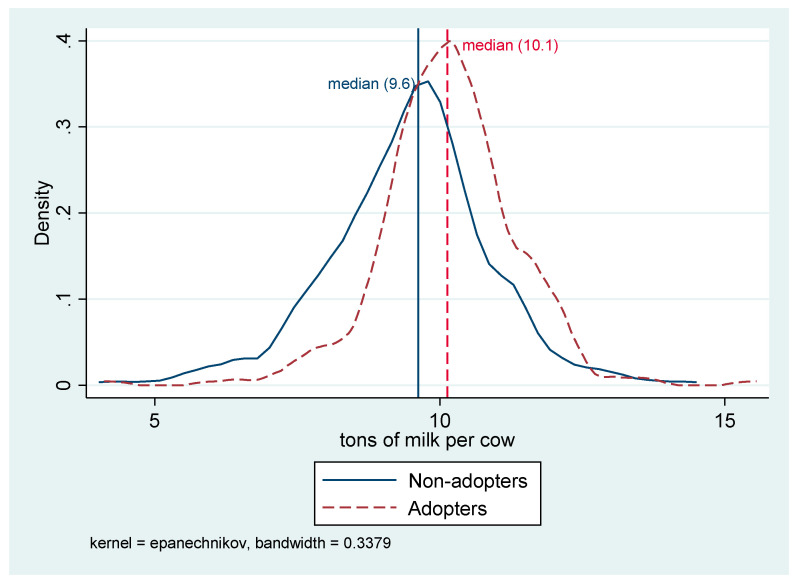
Kernel density estimate of milk production. Source: Our elaborations on survey.

Finally, by simply comparing the 305-d average milk yield per cow, we can see that it is higher for adopters than for non-adopters of about 0.5 tons per cow, evidencing the differences we are interested in and that we will estimate with the regression model. More in detail, Figure 3 shapes non-parametrical curves (Kernel density) of distributions of production index, both for the treated and the control groups. Also with this figure, we can find a confirmation that adopters present a greater concentration of observations towards high values of such index. In fact, the smoothing curve of the adopters shifted more to the right than that of non-adopters and exhibited a higher peak around its median value (Figure 3).

The results of the two stage regressions are reported in the Table 3. Specifically, the first column shows the results of the main regression (i.e., the second stage), which are those we are interested in, while in the second column the first-step probit estimates are reported, for which it was used as dependent variable a dummy indicating the presence/absence of sensor system. The third column shows the coefficients of correction for the main equation from the treatment equation and some tests on the correctness of the specification. 

To illustrate, results of the main regression (column 1) show that, after having (partially) solved the bias due to omitted variables and parity of dimensional conditions of herds, there is a positive effect from using any kind of sensor on farm productivity index. The coefficient of sensors informs us that the intercept increases Y by 0.11 (The applied formula was100 *{*exp* (*β*) − 1}) Then, bringing the inverse of log of coefficient we can calculate a sort of semi-elasticity of 12%, i.e., an ATET of being sensor’ adopter of 1.2 tons of milk per head. The coefficients of both variables, which represent the size of herd, are significant but the first is positive while the second (its square) has negative sign. The interpretation is that, as we expected, the productive scale has a positive effect on the productivity, but at the decreasing rate. In other words, we can see that the scale effect has a non-linear trend, first increasing and then decreasing following a convex curve. 

In the second column, reporting results of the probit regression estimated in the first stage, we can see that the covariates are statistically significant, and the likelihood-ratio test indicates that we can reject the null hypothesis of no correlation between the treatment errors and the outcome errors. Specifically, as the coefficient of farmers’ age has negative sign, it reduces the probability of PLF adoption, confirming that youngest are more prone to introduce innovations in farming. 

Finally, the third column shows some parameters, which are estimated from the two steps procedure and that are needed to make corrections for hazard. Specifically, the estimated correlation between the treatment-assignment errors and the outcome errors, Rho, that is equal −0.36, indicates that un-observables that raise observed productivity tend to occur with un-observables that lower the adoption. In the same column, the negative and significant Lambda coefficient (it also called Mill’s ratio) informs us that without the instrumented correction the ATET measure would have been underestimated. Finally, Sigma is the standard error of the residual in the productivity equation.

## 4. Discussion

This empirical exercise wants to explore the existence on an “adoption effect”, by the estimate of the differences in herd productivity, using individual farms data of 490 livestock farms. Farms are specialized on bovine milk production and are located in one of the most representative areas of Italian dairy farming (in the province of Cremona, Lombardy region). In this regard, we emphasize that farms of this area were highly homogeneous from a production point of view, in terms of genetical capital (breeds) utilized by farms, organization of stalls and management of the herds. The homogeneous production techniques used by farms allow us to make comparisons between those farms who shared the same technological frontier, other than the use of ICT. Moreover, using a two-stage treatment regression technique, we try to solve the selection bias due to unobserved individual heterogeneity and auto-selection of PLF adopters. The main results showed positive relationship between adopter status and the productivity index.

The positive effect of PLF tools on a proxy of farm technical management may be explained by several indirect factors. As stated above, our analysis was conducted regardless the category of tool adopted by the farmers, evidencing a generalized positive effect on milk production. The reasons for this result can be found in the literature about the direct or, more frequently, indirect effect of the main kinds of adopted tools in our survey. Simple examples follow. The data from each milking is one of the most useful to monitor the risk of mastitis [20]. This result, jointly with other sensors for early mastitis detection, allows a positive feedback in maintaining high milk yield in the short time as well as in the whole lactation performance (305-d milk yield). Another example is related to early lameness detection and milk yield. Green et al. (2002) [21] reported how, in clinically lame cows, milk yield was reduced from up to 4 mo before a case of lameness was diagnosed and treated and for the 5 mo after treatment. The consequence on milk yield per 305-d lactation (the variable considered in our study) was a total mean estimated reduction of approximately 360 kg [21]. It is easy arguable how a tool that helps to prevent this problem leads to an improvement in average milk yield in the herd.

Most of the literature evidenced that the improvement in PLF tools efficiency needs to pass through an adequate adoption of new management strategies at herd level. At the beginning, for example, a lot of expectation about robotic milking performance focused essentially on the possible increase in milk production, due to the related increase in milking frequency. Only several years later, the attention was shift on the opportunity to adopt a new herd management to optimize the time use (occupation rate) of each robotic milking stall [22], mainly through an optimal milking interval and a better planning of the number of animals per stall. This led to revise the first estimation about robotic milking investment, because this possible optimization led to an increase in milk revenue of about 6.2% from each milking stall [22].

In one of the few studies attempting to estimate the possible advantages from PLF adoption, [23] reported how productivity did not change after investment in sensor systems on dairy farms. In their study, they evidenced how the investment in sensor systems did not automatically affect the technical performance of the farms [23]. Among several aspects, which resulted not improved as expected, they explained the failure of a reduction on labor requirement as a result of the specific evaluation moment, when the farms were generally focused in an expansion trend rather than on having more free time. Again, a second reason is that the labor saving attained by some automatisms may be counterbalanced by an increase in other tasks such as the data management and interpretation (for example, the alerts checking). Probably, the main effect on labor could be defined as a shift from a simple manpower requirement to a more qualified skill employment.

There are few studies about a possible substitution of capital for labor with the adoption of new technologies in the PLF adoption scenario. One interesting paper was published by Steeneveld et al. (2012) [24] about robotic milking. Even with a 25% higher capital cost, the farms with robotic milking systems did not experience a reduction in labor costs nor an improvement in net outputs [24]. However, the same authors hypothesize that labor savings may occur only after an extended adjustment period, and that a learning effect might be observed over this period.

In the case of milk yield recording devices, a better individual monitoring of the cow could allow a more targeted individual management according to the animal productive ability. One example of this may be the possibility to adequate day by day the amount of supplementary concentrate supplied to the cow to better fit her requirement according to its productivity [25].

Among the systems studied by the North European researchers [26], the introduction of tools for automated mastitis detection was probably the most effective investment to improve the targeted performance (i.e., somatic cells in milk). In the case of devices for mastitis detection, the quick intervention to treat the ill cow may reduce the milk (and financial, [27]) loss in the lactation.

For tools like estrus detection systems, the economic evaluation of the investment must consider the performance in terms of sensitivity and specificity. As reported by Rutten et al. (2014) [4] for an ex ante analysis of activity meters for automated estrus detection, the sensitivity of the tool must be evaluated in light of the current labor required (min/d) for the same task. The different combinations of sensitivity and specificity may affect the final economic evaluation, in terms of marginal financial effect and internal rate of return (IRR) as profitability indicators of the new system. With these assumptions, a correct introduction and use of sensors and systems for automatized estrus detection leads to the most appropriate time for insemination that could contribute to increase individual productivity of a cow ([28]) in her lifetime, and however it indirectly affects the mean of herd productivity, by reducing the rate of non-pregnancy. Finally, an improved quality of data management in the daily routine of the farm could allow a best allocation of the productive factors, firstly labor. Probably, a targeted study on the effects of PLF tools on labor efficiency would be beneficial to a better understanding of the correct way to evaluate the technological expansion strategies of dairy farms. Maybe focusing less on a direct effect of a specific tool on average daily milk per cow, but more on the economic benefit of a better allocation of labor resources.

## 5. Conclusions

Our results confirmed that PLF would help farms to improve their productivity through a better management of herd, suggesting that efforts to increase the adoption of these technologies and the dissemination of adopters’ best practices could contribute to the increase in sector productivity, by improving efficiency in herd management. On the other hand, if there are such efficiency improvements, one wonders why not all farms adopt these technologies? 

Actually, we should consider some other factors. The technology adoption generally increases capital costs, primarily because of higher maintenance costs and depreciation, and it is not so clear when and at which level of investment corresponds a net gain for farm in the adoption of PLF technologies. Moreover, the change from conventional production system to PLF system requires a new management approach and a corresponding change in labor tasks, that could limit the adoption even in the presence of high net gains. In this case, policy interventions addressed at training farmers to make them more confident with such innovative technologies are welcomed. However, more empirical researches are needed to better understand the effects of PLF technologies adoption on the productivity of heterogenous farms and signaling the possible frictions that undermine the potential positives effects at farm level.

## Figures and Tables

**Table 1:** Variables and descriptive statistics.

Variables	Units of Measure	Mean Values			Standard Deviations		
		Control ^1^	Adopters	Total	Control ^1^	Adopters	Total
Used agricultural area (UAA)	hectares	66.6	121.9	96.2	54.9	105.1	89.7
Cows	unit	139.2	232.6	189.1	110.1	143.9	137.3
Total workers	unit	3.6	4.9	4.3	1.3	2.6	2.2
Workers for breeding	unit	1.7	2.4	2.1	0.8	1.5	1.3
Workers for crops	unit	1.9	2.5	2.2	0.8	1.5	1.3
Milk produced	tons/cow	9.5	10.2	9.8	1.4	1.2	1.4

^1^ Control group.

**Table 2:** Number of farmers by age group.

Age Groups	Unit of Measure	Control	Adopters	Total
Less than 30 years	percentage	8.4	7.1	7.7
>=30; <40 years	percentage	11.6	19.2	15.6
>=40; <50 years	percentage	36.4	33.7	35.0
>=50; <60 years	percentage	29.3	32.2	30.8
More than 60 years	percentage	14.2	7.8	10.8

**Table 3:** Results of two stage treatment regression model.

Variables	Log of Milk (cwt/cow/year)	Presence of Sensors (1 = yes, 0 = no)	Hazard
Herd (log of N° of cows)	0.47 ***		
	(0.07)		
Herd^2	−0.04 ***		
	(0.01)		
Sensors (dummy)	0.11 ***		
	(0.05)		
age 30–40 years (dummy)		0.41 *	
		(0.27)	
age 40–50 years (dummy)		0.04	
		(0.24)	
age 50–60 years (dummy)		0.10	
		(0.24)	
age more than 60 years (dummy)		−0.39 *	
		(0.29)	
Cremasco or Cremonese area (dummy)		0.14 *	
		(0.13)	
Size farm (hectares)		0.01 ***	
		(0.00)	
Lambda			−0.05 ***
			(0.03)
Rho			−0.36
Sigma			0.13
Constant	0.92 ***	−0.71 ***	
	(0.18)	(0.24)	

Standard errors in parentheses. *** *p* < 0.01, ** *p* < 0.05, * *p* < 0.1.
